# Targeting TRPC-5 Channel Inhibition to Improve Penile Vascular Function in Erectile Dysfunction

**DOI:** 10.3390/ijms26041431

**Published:** 2025-02-08

**Authors:** Mariam El Assar, Borja García-Gómez, José M. La Fuente, Manuel Alonso-Isa, Juan I. Martínez-Salamanca, Argentina Fernández, Patricia Sosa, Javier Romero-Otero, Leocadio Rodríguez-Mañas, Javier Angulo

**Affiliations:** 1 Fundación para la Investigación Biomédica del Hospital de Getafe, 28905 Getafe, Spain; patricia.sosa@iisgetafe.com; 2 Centro de Investigación Biomédica en Red de Fragilidad y Envejecimiento Saludable (CIBERFES), Instituto de Salud Carlos III, 28029 Madrid, Spain; leocadio.rodriguez@salud.madrid.org; 3 Instituto de Investigación IdiPaz, 28046 Madrid, Spain; 4 Department of Urology, Hospital Universitario 12 de Octubre, Instituto de Investigación Sanitaria Hospital 12 de Octubre (imas12), 28041 Madrid, Spain; gendine@hotmail.com (B.G.-G.); manuelalonsoisa@hotmail.com (M.A.-I.); 5 Serviço de Urologia, Centro Hospitalar e Universitário de Santo António (CHUdSA), 4099-001 Porto, Portugal; lafuentecarvalho@gmail.com; 6 Servicio de Urología, Hospital Universitario Puerta de Hierro, 28222 Majadahonda, Spain; jims09@me.com; 7 Servicio de Histología-Investigación, Unidad de Investigación Traslacional en Cardiología—IRYCIS/UFV, Hospital Universitario Ramón y Cajal, 28034 Madrid, Spain; argentina.fernandez@salud.madrid.org; 8 Servicio de Urología, Hospital Universitario HM Sanchinarro, HM Hospitales, 28050 Madrid, Spain; jromerootero@hotmail.com; 9 Servicio de Geriatría, Hospital Universitario de Getafe, 28905 Getafe, Spain

**Keywords:** canonical transient receptor potential (TRPC) channels, TRPC5, erectile dysfunction, aging, human corpus cavernosum, human penile arteries, endothelial dysfunction, neurogenic relaxation

## Abstract

Canonical transient receptor potential (TRPC) channels contribute to calcium homeostasis, which is involved in penile vascular contractility and erectile dysfunction (ED) pathophysiology. We evaluated the impact of TRPC5 inhibition on endothelial function in penile vascular tissue from aging rats and ED patients and its effect on the relaxant efficacy of PDE5 inhibitors. TRPC inhibitor-induced endothelial and neurogenic relaxations were evaluated in corpus cavernosum (RCC) from a rat model of aging-related ED and in human penile resistance arteries (HPRAs) and corpus cavernosum (HCC) from ED patients and organ donors (NoED). The TRPC5 inhibitor, AC1903, was more effective than TRPC3 and TRPC4 inhibitors in relaxing aged RCC and HCC and HPRA from ED patients. In addition to enhancing endothelial and neurogenic relaxations in RCC from aged animals, AC1903 improved endothelium-dependent relaxation in both HCC and HPRA from ED patients but not in tissues from NoED. Cavernosal expression of TRPC5 was not different between ED and NoED subjects. AC1903 potentiated relaxations to the PDE5 inhibitor, tadalafil, in HCC/HPRA from ED patients. TRPC5 inhibition improved penile vascular function in aged rats and patients with ED. TRPC5 inhibition could be a potential therapeutic target for ED, particularly when combined with PDE5 inhibitors to enhance treatment outcomes.

## 1. Introduction

Erectile dysfunction (ED) is a prevalent and complex condition that primarily affects men over the age of 40, with its occurrence increasing worldwide. This condition is closely associated with cardiovascular disease, diabetes mellitus, hyperlipidemia, and hyperhomocysteinemia among other alterations [1,2]. The prevalence of ED increases with age, with aging itself considered a major independent risk factor for this condition [3,4]. Notably, ED not only impacts sexual health and quality of life [5] but is frequently considered an early indicator of systemic vascular diseases, potentially preceding severe cardiac events [6]. Research has shown a link between ED and an increased risk of both all-cause and cardiovascular-related mortality [7].

The underlying pathogenic mechanisms of ED are rather complex with neurovascular dysfunction as a key causative factor [8]. Studies have reported significantly reduced endothelium-dependent and neurogenic relaxation responses in corpus cavernosum (HCC) strips as well as in penile resistance arteries (HPRAs) derived from patients with ED when compared to organ donors without ED [9,10]. These impairments in vascular function may be partially due to disruptions in the nitric oxide (NO)/cyclic guanosine monophosphate (cGMP) pathway, a key mechanism for penile smooth muscle relaxation and erection [11].

Consistently, the first-line treatment for ED involves enhancing the NO/cGMP pathway through inhibition of type 5 phosphodiesterase (PDE5). However, a substantial subset of patients displays lower response rates to this therapeutic approach, including diabetic men and patients undergoing radical prostatectomy [12], probably due to severe impairment of NO/cGMP pathway [9,13]. This fact highlights the need for alternative therapeutic targets and mechanisms.

Functional enhancement of the stromal interaction molecule (STIM)/Orai calcium entry system has been documented in aging- and ED-related alterations of cavernosal contractility [14,15], especially in diabetic ED in both rat and human penile tissue [16]. This evidence points to a key role of calcium homeostasis in penile vascular pathophysiology. In addition to STIM/Orai system, canonical transient receptor potential (TRPC) channels also appear to contribute to store-operated calcium entry (SOCE) and seem to be involved in the pathophysiology of vascular disorders [17]. In fact, TRPC channels can interact with the STIM/Orai system in regulating calcium concentrations in the vascular system [18,19]. TRPC channels is a subfamily of the TRP channel comprising seven members of non-selective cation channels (TRPC1–7) that are expressed in vascular tissues, including vascular smooth muscle and endothelial cells. They contribute to the regulation of membrane potential and intracellular calcium levels, playing a role in both contraction and relaxation mechanisms within the vasculature [20]. In fact, recent studies point to TRPC channels as potential players in vascular remodeling and disease [21].

However, the role of TRPC channels in vascular dysfunction related to aging and ED remains unclear. Emerging evidence suggests a possible contribution of TRPC channels to ED pathogenesis. For example, TRPC4 channels in corpus smooth muscle cells have been associated with ED in diabetic rats, and downregulation of TRPC4 restored erectile function [22]. Similarly, low androgen levels were found to upregulate TRPC3, TRPC4, and TRPC6 expression in rat penile tissue, contributing to ED [23]. Gene transfer of dominant-negative TRPC6 reduced intracellular calcium levels in human corpus smooth muscle cells and restored erectile function in diabetic rats, suggesting a potential therapeutic approach [24]. Additionally, lysophosphatidylcholine, a component of atherogenic lipoproteins, was shown to increase intracellular calcium in human corpus smooth muscle cells through TRPC6 channel activation, potentially explaining hypercholesterolemia-induced ED [25].

Together, these findings suggest the participation of TRPC channels in ED pathophysiology and could serve as promising therapeutic targets. However, there are currently no data on the effects of TRPC modulation on age- and ED-related vascular dysfunction in penile tissue. Therefore, the aim of the present study was to evaluate the potential of TRPC inhibition as a mechanism for promoting relaxation in penile vascular tissue from aging rats and ED patients. Additionally, the impact of TRPC inhibition on the effectiveness of the inhibitors of PDE5 to induce penile vascular tissue relaxation from men with ED was assessed.

## 2. Results

### 2.1. TRPC5 Inhibitor Caused More Effective Relaxations than TRPC3 and TRPC4 Inhibitors in Corpus Cavernosum from Aged Rats

**Figure 1:**
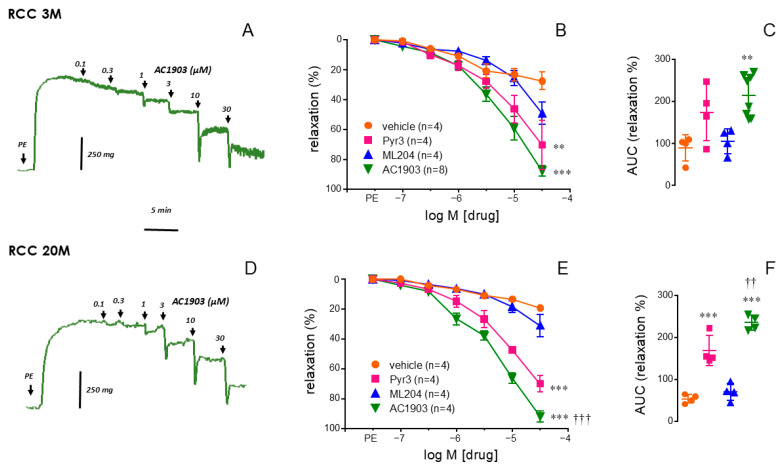
TRPC5 inhibitor outperforms TRPC3 and TRPC4 inhibitors in relaxing aged rat corpus cavernosum. Representative tracings of relaxations induced by TRPC5 inhibitor (AC1903, 0.1 to 30 µM) in corpus cavernosum (RCC) strips from young adult (3-month-old, 3 M, panel (**A**)) and aged (20-month-old, 20 M, panel (**D**)) rats contracted. Panels (**B**,**E**) present concentration–response relaxation curves for vehicle (DMSO 0.001 to 0.3%), the TRPC3 inhibitor (Pyr3), the TRPC4 inhibitor (ML204), and AC1903 in RCC from 3 M rats (**B**) and 20 M rats (**E**). The area under the curve (AUC) of the percentage of relaxation induced by these inhibitors in RCC from 3 M and 20 M rats is represented in (**C**,**F**), respectively. Phenylephrine (PE, 1–10 µM) was used to precontract all tissues. Data are presented as mean ± S.E.M of the relaxation percentage (**B**,**E**) and as individual values and mean ± S.D. of the AUC of relaxation (**C**,**F**). n indicates the number of animals from whom the tissues were collected. ** *p* < 0.01, *** *p* < 0.001 vs. vehicle, and †† *p* < 0.01, ††† *p* < 0.001 vs. Pyr3 by a two-factors ANOVA (**B**,**E**) or by one-factor ANOVA followed by Holm–Sidak’s multiple comparisons test (**C**,**F**).

Increasing concentrations of the TRPC5 inhibitor, AC1903, caused consistent relaxations of phenylephrine (PE)-contracted corpus cavernosum (RCC) strips from young adult (3 M) and aged (20 M) rats (Figure 1A,D). These relaxations were significantly more effective than those caused by the vehicle (*p* < 0.0001) and the TRPC4 inhibitor, ML204 (*p* < 0.0001), in RCC from young rats (Figure 1B), and they were also significantly more effective than those induced by the TRPC3 inhibitor, Pyr3 (*p* < 0.0001), in RCC from aged rats (Figure 1E). This superior efficacy of the TRPC5 inhibitor over the other two TRPC inhibitors to relax RCC strips from aged rats was confirmed when relaxant responses were expressed as the area under the curve (AUC) of relaxation (Figure 1C,F).

### 2.2. Improvement of Cavernosal Neurogenic and Endothelial Relaxant Capacity by the TRPC5 Inhibitor, AC1903, in Aged Rats

**Figure 2:**
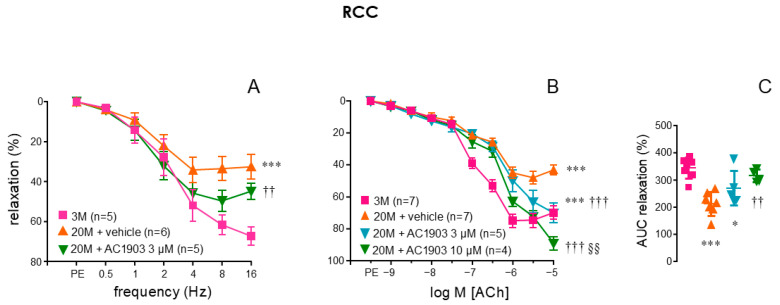
TRPC5 inhibitor, AC1903, improves neurogenic and endothelial relaxation in aged rats. Panel (**A**) illustrates neurogenic nitrergic relaxations elicited by electrical field stimulation (EFS, 0.5 to 16 Hz) in the corpus cavernosum (RCC) from adult (3-month-old, 3 M) and aged (20-month-old, 20 M) rats. The effects of the TRPC5 inhibitor (AC1903, 3 µM) or vehicle (0.3% DMSO) on these responses in aged rats are also shown. Panel (**B**) displays endothelium-dependent relaxations to acetylcholine (ACh, 1 nM to 10 µM) in RCC strips from 3 M and 20 M rats, along with the effects of AC1903 (3 and 10 µM) or vehicle in aged rats. The area under the curve (AUC) for the percentage of endothelium-dependent relaxation following treatment with AC1903 or vehicle is presented in panel (**C**). Data are expressed as mean ± S.E.M of the percentage of relaxation (**A**,**B**) and as individual values and mean ± S.D. of the AUC of relaxation (**C**). n indicates the number of animals from whom the tissues were collected. * *p* < 0.05, *** *p* < 0.001 vs. 3 M, †† *p* < 0.01, ††† *p* < 0.001 vs. 20 M, §§ *p* < 0.01 vs. 20 M with 3 µM AC1903 by a two-factors ANOVA (**A**,**B**) or by one-factor ANOVA followed by Holm–Sidak’s multiple comparisons test (**C**).

Cavernosal strips from aged rats displayed impaired relaxations to EFS when compared to those obtained in young animals (Figure 2A). These neurogenic relaxations were significantly improved when RCC strips from aged rats were pretreated with the TRPC5 inhibitor, AC1903 (3 µM) (*p* = 0.0075) (Figure 2A). In fact, these nitrergic relaxations of aged RCC strips obtained in the presence of AC1903 were not significantly different (*p* = 0.2382) from those displayed by RCC strips from young animals (Figure 2A).

Aging was also associated with an impairment of endothelial function as evidenced by the significant reduction in the relaxations induced by ACh (1 nM to 10 µM) in RCC strips from aged rats (*p* < 0.0001) (Figure 2B). Treatment of RCC strips from aged rats with AC1903 at 3 µM concentration significantly enhanced ACh-induced responses (*p* = 0.0006) that were further improved when increasing the concentration of AC1903 to 10 µM (*p* < 0.0001 vs. 20 M + vehicle and *p* = 0.0073 vs. 20 M + AC1903 3 µM) (Figure 2B). In fact, after treatment with 3 µM AC1903, RCC strips from aged rats still presented a significant impairment of endothelial relaxations with respect to young animals (*p* < 0.0001), but no significant impairment was observed after treating aged strips with 10 µM (*p* = 0.1578) (Figure 2B). Expressing the results as the AUC of endothelial relaxations yielded confirmation of these effects (Figure 2C). In contrast, treating RCC strips from young animals with the activator of TRPC5, BTD (10 µM), resulted in a reduction in endothelial relaxations (Supplementary Figure S1).

### 2.3. TRPC5 Inhibitor, AC1903, Drives Relaxation of Human Penile Vascular Tissues

Clinical data from human subjects included in the study are summarized in Table 1. Compared to organ donors without notice of ED (NoED), ED patients were older and exhibited more cardiovascular risk factors, including diabetes, dyslipidemia, and smoking habits, as well as a higher prevalence of cardiovascular disease. Additionally, a significant number of ED patients previously underwent radical pelvic surgery (11 prostatectomy and 1 cystectomy).

**Figure 3:**
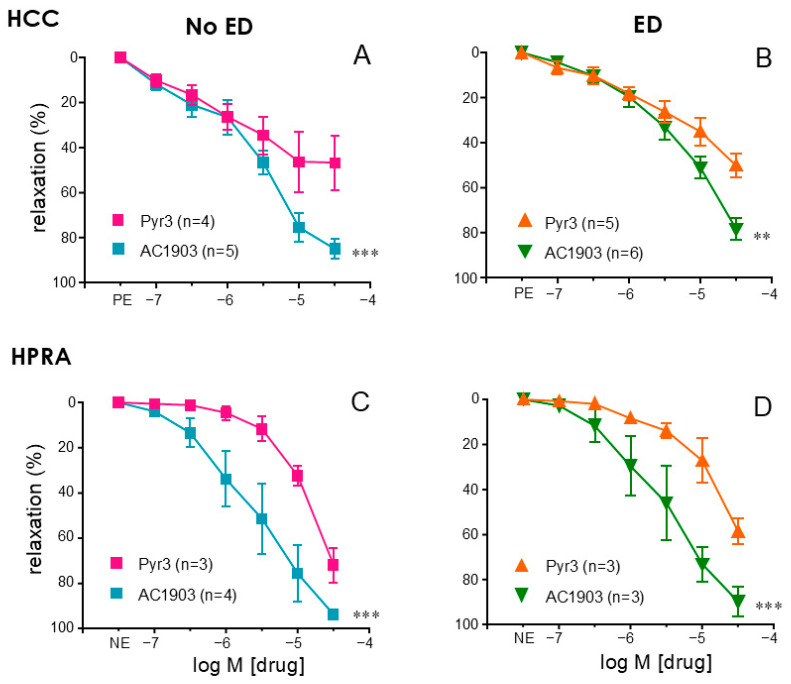
TRPC5 inhibition promotes effective relaxations in human penile vascular tissue. Upper panels show relaxation responses to increasing concentrations (0.1 to 30 µM) of the TRPC3 inhibitor (Pyr3), or the TRPC5 inhibitor AC1903 in human corpus cavernosum (HCC) from organ donors without erectile dysfunction (NoED, panel (**A**)) and from patients with erectile dysfunction (ED, panel (**B**)). The same responses were evaluated in human penile resistance arteries (HPRAs) from NoED subjects (**C**) and in subjects with ED (**D**). HCC strips were precontracted with phenylephrine (PE, 1–10 µM), while norepinephrine (NE, 1–10 µM) was used to precontract HPRA segments. Data are expressed as mean ± S.E.M of the relaxation percentage. n represents patients’ number from whom the tissues were obtained. ** *p* < 0.01 and *** *p* < 0.001 vs. Pyr3 by a two-factors ANOVA.

Human cavernosal strips contracted with PE were consistently relaxed by adding increasing concentrations of the TRPC5 inhibitor, AC1903 (0.1 to 30 µM). These relaxations were significantly more effective than those caused by the TRPC3 inhibitor, Pyr3 (0.1 to 30 µM). These effects were observed in cavernosal strips from both subjects without notice of ED (NoED) (*p* = 0.0009) (Figure 3A) and patients with ED (*p* = 0.0015) (Figure 3B). In the same way, the capacity of AC1903 to cause relaxations in human penile resistance arteries (HPRA) contracted with norepinephrine (NE) was significantly superior to that of Pyr3 in arteries from NoED subjects (*p* = 0.0004) (Figure 3C) as well as from patients with ED (*p* = 0.0003) (Figure 3D).

### 2.4. Endothelial Relaxation of Human Penile Vasculature from ED Patients Is Improved by the TRPC5 Inhibitor, AC1903

**Figure 4:**
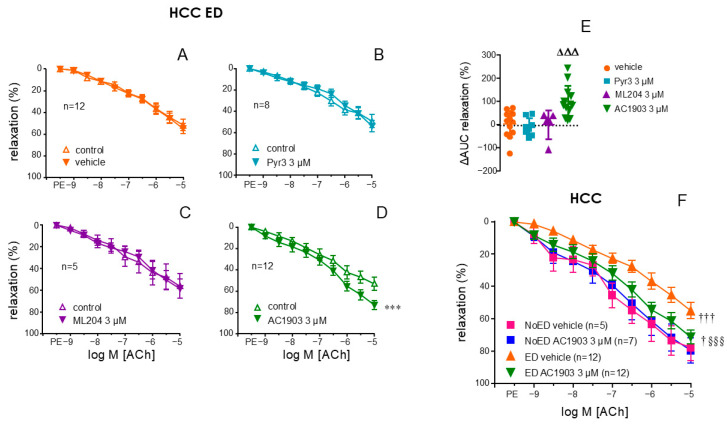
TRPC5 inhibitor AC1903 enhances endothelial-mediated relaxation in human corpus cavernosum (HCC) of ED patients. Panels A-D illustrate the effects of vehicle (DMSO 0.3%, (**A**)), the TRPC3 inhibitor (Pyr3, 3 µM, (**B**)), the TRPC4 inhibitor (ML204, 3 µM, (**C**)), and the TRPC5 inhibitor (AC1903, 3 µM, (**D**)) on endothelium-dependent relaxation responses to acetylcholine (ACh, 1 nM to 10 µM) in HCC from patients with ED. Panel (**E**) summarizes the change in the area under the curve (ΔAUC) of endothelial relaxation relative to control conditions induced by the treatments in HCC from ED patients. Panel (**F**) compares the effect of AC1903 or vehicle on endothelium-dependent relaxations to ACh in HCC strips from organ donors without notice of ED (NoED) and from ED patients. Phenylephrine (PE, 1–10 µM) was used to precontract HCC strips. Data are presented as mean ± S.E.M of the relaxation percentage (**A**–**D**,**F**) and as individual values and mean ± S.D. of the ΔAUC of relaxation (**E**). n represents patients’ number from whom the tissues were collected. *** *p* < 0.001 vs. control by a two-factors ANOVA test, ΔΔΔ *p* < 0.001 vs. vehicle by one-factor ANOVA followed by Holm–Sidak’s multiple comparisons test, † *p* < 0.05, ††† *p* < 0.001 vs. NoED, §§§ < 0.001 vs. ED by a two-factors ANOVA test.

In cavernosal strips from patients with ED, a significant improvement of endothelium-dependent ACh-induced relaxations was obtained after treating the strips with the TRPC5 inhibitor, AC1903 (3 µM) (*p* < 0.0001) (Figure 4D). This was not produced by the treatments with the vehicle (0.3% DMSO) (Figure 4A), the TRPC3 inhibitor, Pyr3 (3 µM) (Figure 4B) or the TRPC4 inhibitor, ML204 (3 µM) (Figure 4C). In fact, AC1903 was the only treatment able to induce a significant increment in AUC (ΔAUC) of relaxation over the AUC in control conditions (*p* = 0.0008) (Figure 4E). Furthermore, a potentiating effect by AC1903 on endothelial relaxation was not produced in cavernosal strips from NoED subjects. In fact, treatment with AC1903 reversed, although not completely (*p* = 0.0163 vs. NoED), the impairment of endothelial relaxations displayed by cavernosal tissue from ED patients (Figure 4F).

**Figure 5:**
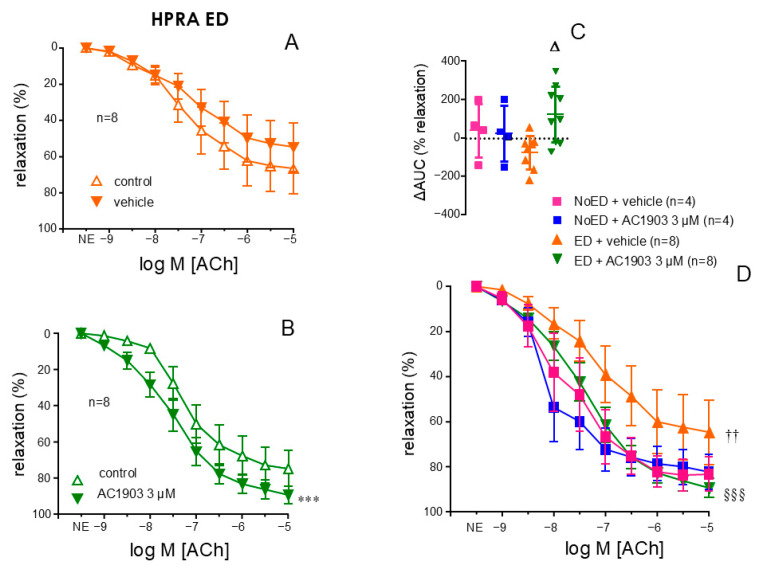
TRPC5 inhibitor AC1903 enhances endothelial-mediated relaxation in human penile resistance arteries (HPRAs) of ED patients. Left panels show the effect of vehicle (DMSO, 0.3%, panel (**A**)), and the TRPC5 inhibitor (AC1903, 3 µM, panel (**B**)) on endothelial relaxations induced by acetylcholine (ACh, 1 nM to 10 µM) in HPRA from ED patients. Panel (**C**) summarizes the change in the area under the curve (ΔAUC) of endothelial relaxation relative to control conditions induced by AC1903 and the vehicle in HPRA from organ donors without notice of ED (NoED) and ED patients. Panel (**D**) displays the effects of AC1903 or vehicle on endothelium-dependent relaxations to ACh in HPRA segments from No ED subjects and from ED patients. Norepinephrine (NE, 1–10 µM) was used to precontract HPRA segments. Data are presented as mean± S.E.M of the percentage of relaxation (**A**,**B**,**D**) and as individual values and mean ± S.D. of the ΔAUC of relaxation (**C**). The number of patients from whom tissues were obtained is denoted by n. *** *p* < 0.001 vs. control by a two-factors ANOVA test, Δ *p* < 0.05 vs. vehicle by one-factor ANOVA followed by Holm–Sidak’s multiple comparisons test, †† *p* < 0.01 vs. NoED, §§§ < 0.001 vs. ED by a two-factors ANOVA test.

Analogously, AC1903 (3 µM) or the vehicle (0.3% DMSO) did not cause significant modifications in endothelial relaxations in HPRA from NoED subjects (Figure 5D). Nevertheless, the treatment with AC1903 (Figure 5B) but not the vehicle (Figure 5A) resulted in a significant improvement of endothelial vasodilation of penile arterial segments from patients with ED (*p* < 0.0001) as also evidenced by the significant increase in ΔAUC of relaxation (Figure 5C). Moreover, the treatment with AC1903 (3 µM) reversed the impairment of endothelial vasodilation related to the presence of ED (*p* = 0.0260) (Figure 5D).

**Figure 6:**
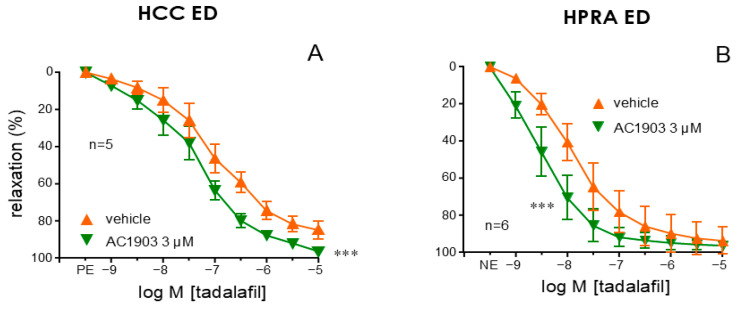
Inhibition of TRPC5 increased the efficacy of PDE5 inhibition in relaxing the penile vasculature of patients with ED. Panel (**A**,**B**) illustrates the effect of vehicle (DMSO 0.3%) and theTRPC5 inhibitor (AC1903, 3 µM) on endothelial relaxations induced by the PDE5 inhibitor sildenafil (1 nM to 10 µM) in the HCC (**A**) and in penile resistance arteries (HPRAs) from ED patients (**B**). Phenylephrine (PE, 1–10 µM) was used to precontract HCC strips while HPRA segments were precontracted with norepinephrine (NE, 1–10 µM). Data are presented as mean ± S.E.M of the relaxation percentage. The number of patients from whom tissues were obtained is denoted by n. *** *p* < 0.001 vs. vehicle by a two-factors ANOVA.

The PDE5 inhibitor, tadalafil (1 nM to 10 µM), promoted relaxation of cavernosal strips precontracted with PE and penile arteries precontracted with NE obtained from patients with ED. These relaxant responses to tadalafil were significantly potentiated when cavernosal strips (*p* < 0.0001) (Figure 6A) or penile arteries (*p* = 0.0008) (Figure 6B) were previously treated with the TRPC5 inhibitor, AC1903 (3 µM).

### 2.5. TRPC5 Protein Expression Is Detected in Cavernosal Tissue from NoED Subjects and from Patients with ED

**Figure 7:**
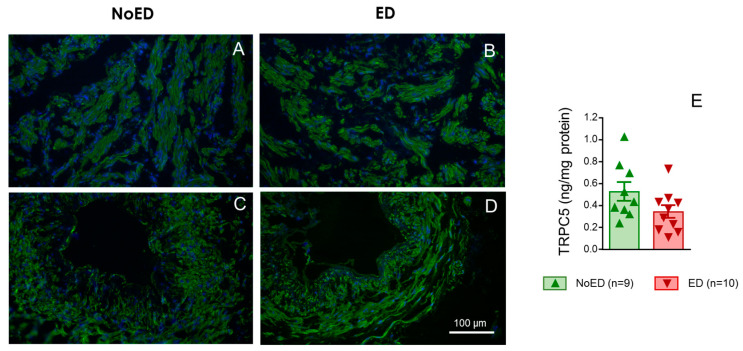
TRPC5 expression identified in human cavernosal tissue and penile arteries. Left panels show representative immunofluorescence images showing TRPC5 detection (green fluorescence) in cryosections of the corpus cavernosum (HCC) and penile resistance arteries (HPRAs) from individuals without erectile dysfunction (NoED, panels (**A**,**C**)) and patients with erectile dysfunction (ED, panels (**B**,**D**)). Nuclei are counterstained in blue. Panel (**E**) shows the quantification of TRPC5 protein levels in human cavernosal homogenates from NoED subjects and ED patients. Data are expressed as individual values and mean ± S.E.M. of the nanograms of TRPC5 normalized to the tissue’s protein content. n indicates number of subjects. No significant differences were observed.

Immunofluorescence assays revealed consistent expression of TRPC5 protein in cavernosal tissues from either NoED subjects (Figure 7A) or patients with ED (Figure 7B). TRPC5 was clearly immunodetected in cavernosal smooth muscle cells (Figure 7A,B) but also in the muscle layer of penile arteries from both ED patients and NoED subjects (Figure 7C,D). Determination of TRPC5 protein content in human cavernosal homogenates confirmed the expression of this cation channel in cavernosal tissue. TRPC5 content in corpus cavernosum from patients with ED was not significantly different to that determined in tissues from NoED subjects (*p* = 0.1122) (Figure 7E).

## 3. Discussion

The results point to the inhibition of TRPC5 as a strategy to relieve the impairment of cavernosal relaxation related to aging in rats and to improve endothelial function and response to conventional therapy in human penile vascular tissue. The beneficial effect of TRPC5 inhibition on cavernosal function in ED is produced despite the lack of modification in the expression of TRPC5 channels in cavernosal tissues with this condition.

Aging is a main risk factor for the development of ED [3,4]. In fact, it has been previously shown that Sprague Dawley rats display reduced erectile responses at 20 months of age [26]. This means that the improving effects of TRPC inhibition on cavernosal relaxation, reported here, were evidenced in a rat model of aging-related ED. In corpus cavernosum from 20-month-old rats, impairments of endothelial and neurogenic relaxations have been previously demonstrated [15,27], which were further confirmed in the present study. The rationale for evaluating TRPC non-selective calcium channels as potential targets in this model is grounded in the fact that the entry calcium system STIM/Orai contributed to aging- and ED-related penile pathophysiology [14,16]. TRPCs participate together with Orai channels in SOCE, contributing to calcium homeostasis [28]. Moreover, a functional link between STIM/Orai system and TRPCs has been proposed since the Orai channel inhibitor, YM-58483, also produces inactivation of TRPC3 and TRPC5 channels [29]. Then, we aimed to assess if the previously evidenced functional effects exerted by YM-58483 on cavernosal contractility could be reproduced by specifically targeting TRPC3 and TRPC5 as well as the closely related to TRPC5, TRPC4. The selective inhibitors Pyr3, ML204 and AC1903 were used for evaluating the effects of TRPC3, TRPC4 and TRPC5, respectively [30,31,32]. TRPC3 and TRPC5 inhibitors were able to consistently relax precontracted cavernosal strips from young and old rats, but the inhibitor of TRPC5 was significantly more potent than Pyr3 in tissues from old ones. This evidence suggests that TRPC4, despite its structural similarity to TRPC5 [33], does not seem to participate in regulating contractile tone in rat corpus cavernosum. Moreover, TRPC5 channels appear to be more relevant contributors to contractile tone than TRPC3, especially in aging conditions. This evidence obtained in rat cavernosal tissue was confirmed in human penile vascular tissues since TRPC5-inhibition-induced relaxations were significantly more potent than those caused by TRPC3 inhibition in human penile cavernosal and arterial preparations in tissues coming from NoED subjects or from patients with ED. Considering that the relative contribution of different players to the finetuning of calcium levels and smooth muscle contraction may not necessarily be the same across species or vascular territories, the confirmation in human penile vascular tissues of the effects of TRPC5 inhibitor is highly relevant.

The ability of AC1903 to potently reverse adrenergic tone led us to evaluate the effects of TRPC5 inhibition on neurogenic and endothelial relaxation of cavernosal tissues from aged rats. A significant improvement of both neurogenic and endothelial relaxations was obtained after treating the cavernosal tissues from aged rats with 3 µM AC1903. This indicates that TRPC5 inhibition counteracts the reduction in relaxant responses, which is a feature of different models of ED [16,34,35,36]. Moreover, increasing the concentration of AC1903 to 10 µM results in further improvement of endothelial relaxations in aged corpus cavernosum, suggesting pharmacological effects. This concept is further supported by the fact that activating TRPC5 receptors with BTD [37] results in the opposite effects, i.e., it caused a reduction in endothelial relaxations in cavernosal strips from young rats.

Again, the ability of TRPC5 inhibition to improve physiological relaxations such as endothelial relaxations was not only observed in the rat model of aging-related ED but also in penile vascular tissues from men with ED. This effect was not achieved with TRPC3 or TRPC4 inhibitors, indicating a specific functional impact of TRPC5 inhibition on relaxations. The improvement of endothelial responses by AC1903 was observed in cavernosal tissue and penile arteries from patients with ED but not in penile tissues from NoED subjects. This could be due to an ED-related increase in TRPC5 expression in penile tissues, but it seems not to be the case. Immunoassays revealed a consistent expression of TRPC5 in smooth muscle from both cavernosal and penile arterial tissues from both NoED subjects and ED patients and determination of cavernosal content of TRPC5 channel protein by ELISA indicated that the expression of TRPC5 is not significantly modified by ED condition. We cannot discard ED-related upregulation of TRPC5 activity but the lack of effect of TRPC5 inhibitor on NoED subjects could also be related to the fact that the relaxant responses in these tissues are presumably preserved.

The improving effects of TRPC5 inhibition on endothelial function has potential therapeutic implications since endothelial dysfunction is involved the pathophysiology of ED [12], and the impairment of endothelial function has been evidenced in corpus cavernosum and penile arteries with aging [38] and with ED [9,13]. However, an additional therapeutic implication arose when AC1903 enhanced the relaxant capacity of a PDE5 inhibitor, the first-line therapy for ED, in corpus cavernosum and penile arteries from ED patients. PDE5 inhibitors display good efficacy in the treatment of ED, but there is a significant percentage of patients who do not respond to this therapeutic approach [12,39]. Thus, combination therapies increasing the pharmacological efficacy of PDE5 inhibitors could be of help to increase the therapeutic response of men with ED.

AC1903 has been designed and characterized as a selective TRPC5 inhibitor [32], and it is considered as an adequate pharmacological tool to study TRPC5 channels [40]. Furthermore, through its ability to inhibit TRPC5, AC1903 has been demonstrated to produce in vivo therapeutic effects in different animal models of kidney disease [41], an improving effect shared by other TRPC5 inhibitors in rats [42]. In fact, a TRPC5 inhibitor has entered a phase 2 clinical trial for treating severe forms of kidney disease [43], supporting consideration of TRPC5 as a therapeutic target of clinical usefulness.

Although the results strongly suggest the involvement of TRPC5 inhibition in the improvement of cavernosal relaxations in ED conditions by AC1903, the existence of research proposing additional targets for this compound should be acknowledged. In fact, it has been suggested to also inhibit TRPC3 and TRPC4 channels [44]. This probably does not apply for our study, since specific TRPC3 and TRPC4 inhibitors failed to exert improving effects.

Strengths of the present study include the functional evaluation of animal models and human tissue from both healthy individuals and patients with ED. Although we included tissues from patients with different types of ED, limitations in the translatability of the present results are related to the complexity of ED pathophysiology, which involves very different scenarios, from the psychogenic to the hormonal, neurogenic and vascular components. This means that TRPC5 inhibition cannot be equally efficient in all conditions.

## 4. Materials and Methods

### 4.1. Experimental Animals

Adult (3-month-old, 3 M, *n* = 22) and aged (20-month-old, 20 M, *n* = 28) male Sprague Dawley rats were bred in the Animal Facilities of the Hospital Ramón y Cajal and housed under 12-h light/dark cycles with unrestricted access to food and water until the start of experimental procedures. All animal experiments complied with the Declaration of Helsinki and with the Guide for the Care and Use of Laboratory Animals, as adopted and promulgated by National Institutes of Health, following European regulations. The procedures were approved by the Ethics Committee for Animal Experimentation of the Hospital Ramón y Cajal (PROEX 183.1/23). All studies were reported in accordance with the ARRIVE guidelines for reporting experiments involving animals. Experiments with adult (3 M) and aged (20 M) rats were intercalated to avoid possible sequence-dependent bias.

#### Functional Evaluation of Rat Cavernosal Tissues

At specific ages (3 M or 20 M), rats were weighed and anesthetized via intraperitoneal injection of diazepam (5 mg/kg) and ketamine (90 mg/kg). Under deep anesthesia, humane euthanasia was performed through exsanguination, and the penises were immediately removed for functional studies. The tunica albuginea was carefully incised longitudinally, and two strips of corpus cavernosum (RCC) were dissected from each penis. Cavernosal strips were mounted on force transducers in 8 mL organ baths filled with Krebs–Henseleit solution (KHS) maintained at 37 °C. The KHS had the following composition (mM): NaCl 119, KCl 4.6, CaCl_2_ 1.5, MgCl_2_ 1.2, NaHCO_3_ 24.9, glucose 11, KH_2_PO_4_ 1.2, EDTA 0.027; it was continuously bubbled with 95% O_2_/5% CO_2_ (pH 7.4). The strips were subjected to a resting tension of 0.3 g and allowed to equilibrate for 60 min. Following this period, tissues were exposed to 125 mM K^+^, and contraction was measured. Once a stable contraction was achieved with phenylephrine (PE, 1–3 µM), relaxation responses were obtained by exposing the strips to increasing concentrations (0.1 to 30 µM) of TRPC antagonists, Pyr3 (TRPC3), ML204 (TRPC4), and AC1903 (TRPC5). Endothelium-dependent relaxations were evaluated by adding increasing concentrations of acetylcholine (ACh, 1 nM to 10 µM) on PE-contracted strips treated with vehicle (DMSO 0.3%) or AC1903 (3 and 10 µM).

Electrical field stimulation (EFS) was applied to RCC strips by means of two platinum electrodes placed at both sides of the tissue and connected to a current stimulator (Cibertec, Madrid, Spain). Parameters of EFS were as follows: 75 mA, 0.5 ms for 20 s. Neurogenic relaxations in response to EFS (0.5 to 16 Hz) were assessed in RCC strips pretreated with guanethidine (30 µM) and atropine (0.1 µM) and contracted with PE (1–3 µM). The protocols for EFS-induced neurogenic relaxations in RCC were detailed in previous studies [9,16]. Relaxation responses induced by ACh and EFS were performed under control conditions and following a 20-min exposure to the vehicle (DMSO 0.3%), Pyr3 (3 µM) or AC1903 (3 µM).

### 4.2. Human Tissues

Human corpus cavernosum (HCC) biopsies were obtained from men with ED (*n* = 45) who gave written informed consent at the time of penile prosthesis implantation as well as from organ donors with no reported history of ED (NoED, *n* = 15) at the time of organ collection for transplantation, after obtaining written informed consent by their relatives. Adult patients undergoing penile implantation surgery due to organic ED (including neurogenic, vascular or any other type of organic ED) were included in the study. Patients with infectious diseases and/or undergoing prosthesis re-implantation were excluded. Protocols and consent forms were approved by the Ethics Committee at the Hospital Universitario Doce de Octubre, Madrid, Spain (Ethics Approval procedure 16/045) and Hospital Geral Santo Antonio, Porto, Portugal (2015.210(174-DEFI/156-CES)), where the tissues were collected. Tissues were maintained at 4 °C to 6° C in M-400 solution (composition per 100 mL: mannitol, 4.19 g; KH_2_PO_4_, 0.205 g; K_2_HPO_4_•3H_2_O, 0.97 g; KCl, 0.112 g; NaHCO_3_, 0.084 g; pH 7.4) until use, which was between 16 and 24 h from extraction [10,12,16].

#### 4.2.1. Functional Evaluation of HCC

Strips of cavernosal tissue (3 mm × 3 mm × 7 mm) were placed in 8 mL organ baths filled with KHS, maintained at 37°C, and continuously aerated with a 95% O_2_/5% CO_2_ mixture to stabilize the pH at 7.4. Strips were tied to force transducers for isometric tension recording as previously described [9,10,14]. Each tissue strip was incrementally stretched to optimal isometric tension, which was determined by maximal contractile response to 1 µM PE. The preparations were then exposed to high K^+^ concentration (125 mM), and the contractile response was measured. Relaxation responses were evaluated in HCC strips precontracted with PE (1–3 µM). Endothelium-dependent relaxations were evaluated by adding increasing concentrations of ACh (1 nM to 10 µM) in control conditions or after treating for 20 min with vehicle (DMSO 0.3%), Pyr3, ML204 or AC1903 (all at 3 µM). The effects of the treatment with vehicle or AC1903 (3 µM) on relaxation responses to the PDE5 inhibitor, tadalafil (1 nM to 10 µM) were also evaluated.

#### 4.2.2. Assessment of Functional Responses in Human Penile Resistance Arteries (HPRAs)

Small helicine arteries of the penis (lumen diameter 150–400 µm) were dissected from HCC specimens by carefully excising the surrounding cavernosal tissue. Arterial segments measuring 1.7–2.0 mm in length were mounted onto microvascular wire myographs (Danish MyoTechnology; Aarhus, Denmark) for isometric tension recordings [10]. The vessels were equilibrated for 30 min in KHS at 37° C and continuously aerated with a 95% O_2_/5% CO_2_ gas mixture to sustain a pH of 7.4. The internal diameter of each vascular segment was measured when relaxed in situ under a transmural pressure of 100 mm Hg (L_100_). The arteries were adjusted to an internal diameter corresponding to 90% of L_100_, at which the force generation was close to maximum. The preparations were then exposed to high K+ and the contractile response was measured. HPRA segments were excluded from the study if they failed to generate tension equivalent to 100 mmHg. Endothelial vasodilations to ACh and vasorelaxant responses to tadalafil were evaluated in arterial segments precontracted with NE (1–3 µM) and treated with vehicle or AC1903 (3 µM).

A schematic representation of the dissection of rat and human penile tissues for functional assays is provided in Supplementary Figure S2.

### 4.3. Immunofluorescence Assays

Cavernosal strips and penile arteries were cleaned of blood and immersed in saccharose at increasing percentages (10% to 30% *w/v*). After 1 h at the higher percentage of saccharose, tissues were embedded in OCT and stored at −80 °C until use for immunofluorescence assays. Then, OCT blocks were sectioned into 6 μm thick transverse slices using a cryostat and mounted onto polylysine-coated glass slides. For the immunodetection of TRPC5, OCT was removed from the sections, which were then fixed in a mixture of acetone and methanol to eliminate autofluorescence. Sections were then incubated with monoclonal mouse antibody against TRPC5 (Invitrogen by ThermoFisher Scientific, Waltham, MA, USA, cat.# MA5-27657, 1:250 dilution) overnight at 4 °C. After washout in phosphate-buffered saline plus 0.3% Triton X-100, the sections were incubated for 1 h at room temperature with a secondary Alexa Fluor 488-conjugated goat anti-mouse antibody (dilution 1:250; SouthernBiotech, Birmingham, AL, USA, cat.# 1031-30) and counterstained with diamidino-2-phenylindole (DAPI; Biorbyt, Cambridge, UK) to visualize the nuclei. The sections were prepared for visualization and examined under a fluorescence microscope (Olympus BX51, Olympus Corporation, Tokyo, Japan).

### 4.4. TRPC5 Protein Content in Human Cavernosal Tissues

Human cavernosal tissues were rapidly immersed in liquid nitrogen for freezing and kept at −80 °C until protein extraction. Proteins were obtained by homogenizing the cavernosal tissue using T-PER extraction reagent (Pierce Biotechnology, Inc., Rockford, IL, USA) in a TissueLyser LT homogenizer (Qiagen Iberia, S.L. Barcelona, Spain) according to the manufacturer’s recommendations. Protease inhibitor cocktail (1×) was also added (Roche Diagnostics, Indianapolis, IN, USA). Total protein content in homogenates was determined by the bicinchoninic acid (BCA) method (ThermoFisher). TRPC5 protein content was quantified by means of a specific ELISA kit (FineTest, Wuhan, China, cat.# EH5212) following instructions indicated by manufacturer. Values of TRPC5 content were normalized by total protein concentrations.

### 4.5. Data Analysis

Data in concentration–response curves are expressed as the mean ± S.E.M. of the percentage of maximum relaxation obtained by adding papaverine (0.1 mM) at the end of the experiment. The area under the curve (AUC) of the relaxation responses was calculated by determining the sum of percentages of relaxation along the concentration–response curve. The increment in AUC (ΔAUC) was obtained by calculating the difference in the AUC after respective treatment with respect to AUC determined in control conditions. Data of AUC and ΔAUC are presented as individual values and mean ± S.D. Patient data were compared by Mann–Whitney U-test (age) or Fisher’s exact test (categorical variables). Complete concentration–response curves were compared by two-factors ANOVA. When more than two curves were compared, Bonferroni’s correction was applied. All other data were compared by Mann–Whitney U-test or one-factor ANOVA followed by Holm–Sidak’s test for multiple comparisons. Differences were considered significant when probability (*p*) was <0.05.

## 5. Conclusions

TRPC5 inhibition improves penile vascular function in an animal model of aging-related ED and in tissues from patients with ED. This is observed despite the lack of TRPC5 upregulation in cavernosal tissue from ED patients. Considering the key role of the impairment of penile vascular tissue relaxation in the pathophysiology of ED, TRPC5 inhibition could be considered a potential therapeutic target in the management of ED, including pharmacological combination with PDE5 inhibitors to increase therapeutic response.

## Figures and Tables

**Table 1:** Characteristics of human subjects.

	NoED	ED	*p* Value
n	15	45	
Age (years)	50.5 ± 4.1	59.1 ± 1.4	***0.0201***
Diabetes (%)	0 (0.0)	15 (33.3)	***0.0318***
Dyslipidemia (%)	0 (0.0)	16 (35.6)	***0.0060***
Hypertension (%)	2 (12.5)	19 (42.2)	0.0609
Obesity (%)	0 (0.0)	9 (20.0)	0.0955
Smoking habit (%)	0 (0.0)	26 (57.8)	***<0.0001***
Cardiovascular disease (%)	1 (6.3)	15 (33.3)	***0.0499***
Peyronie’s disease (%)	0 (0.0)	3 (6.7)	0.5660
BPH (%)	1 (6.3)	4 (8.9)	1.0000
Pelvic surgery (%)	0 (0.0)	12 (26.7)	***0.0269***
Neurological alteration (%)	0 (0.0)	3 (6.7)	0.5660
Respiratory disease (%)	0 (0.0)	6 (13.3)	0.3214

BPH: benign prostate hyperplasia; ED: erectile dysfunction; NoED: organ donors without notice of ED. n represents the number of subjects. The Mann–Whitney U-test was used to compare age, while Fisher’s exact test was applied to assess differences in other variables between the two groups. Significant differences are highlighted in bold plus italics.

## Data Availability

The data presented in this study are available on request from the corresponding authors. The data are not publicly available due to data protection policy.

## Supplementary Materials

The following supporting information can be downloaded at: https://www.mdpi.com/article/10.3390/ijms26041431/s1.

## References

[1] DeLay K.J., Haney N., Hellstrom W.J. (2016). Modifying Risk Factors in the Management of Erectile Dysfunction: A Review. World J. Mens. Health.

[2] Salvio G., Ciarloni A., Cordoni S., Cutini M., Delli Muti N., Finocchi F., Firmani F., Giovannini L., Perrone M., Balercia G. (2022). Homocysteine levels correlate with velocimetric parameters in patients with erectile dysfunction undergoing penile duplex ultrasound. Andrology.

[3] Echeverri Tirado L.C., Ferrer J.E., Herrera A.M. (2016). Aging and Erectile Dysfunction. Sex. Med. Rev..

[4] Kaya E., Sikka S.C., Kadowitz P.J., Gur S. (2017). Aging and sexual health: Getting to the problem. Aging Male.

[5] McMahon C.G. (2019). Current diagnosis and management of erectile dysfunction. Med. J. Aust..

[6] Blick C., Ritchie R.W., Sullivan M.E. (2016). Is Erectile Dysfunction an Example of Abnormal Endothelial Function?. Curr. Vasc. Pharmacol..

[7] Kessler A., Sollie S., Challacombe B., Briggs K., Van Hemelrijck M. (2019). The global prevalence of erectile dysfunction: A review. BJU Int..

[8] Giuliano F. (2008). New horizons in erectile and endothelial dysfunction research and therapies. Int. J. Impot. Res..

[9] Angulo J., González-Corrochano R., Cuevas P., Fernández A., La Fuente J.M., Rolo F., Allona A., Sáenz de Tejada I. (2010). Diabetes Exacerbates the Functional Deficiency of NO/cGMP Pathway Associated with Erectile Dysfunction in Human Corpus Cavernosum and Penile Arteries. J. Sex. Med..

[10] El Assar M., La Fuente J.M., Sosa P., Fernández A., Pepe-Cardoso A.J., Martínez-Salamanca J.I., Rodríguez-Mañas L., Angulo J. (2024). PKC Inhibition Improves Human Penile Vascular Function and the NO/cGMP Pathway in Diabetic Erectile Dysfunction: The Role of NADPH Oxidase. Int. J. Mol. Sci..

[11] Burnett A.L. (2006). The role of nitric oxide in erectile dysfunction: Implications for medical therapy. J. Clin. Hypertens..

[12] Mitidieri E., Cirino G., d’Emmanuele di Villa Bianca R., Sorrentino R. (2020). Pharmacology and perspectives in erectile dysfunction in man. Pharmacol. Ther..

[13] Martínez-Salamanca J.I., La Fuente J.M., Fernández A., Martínez-Salamanca E., Pepe-Cardoso A.J., Carballido J., Angulo J. (2015). Nitrergic function is lost but endothelial function is preserved in the corpus cavernosum and penile resistance arteries of men after radical prostatectomy. J. Sex. Med..

[14] Sevilleja-Ortiz A., El Assar M., García-Rojo E., Romero-Otero J., García-Gómez B., Fernández A., Medina-Polo J., La Fuente J.M., Rodríguez-Mañas L., Angulo J. (2020). Enhanced Contribution of Orai Channels to Contractility of Human Penile Smooth Muscle in Erectile Dysfunction. J. Sex. Med..

[15] Sevilleja-Ortiz A., El Assar M., García-Rojo E., García-Gómez B., Fernández A., Sánchez-Ferrer A., La Fuente J.M., Romero-Otero J., Rodríguez-Mañas L., Angulo J. (2021). Ageing-induced hypercontractility is related to functional enhancement of STIM/Orai and upregulation of Orai 3 in rat and human penile tissue. Mech. Ageing Dev..

[16] Sevilleja-Ortiz A., El Assar M., García-Gómez B., La Fuente J.M., Alonso-Isa M., Romero-Otero J., Martínez-Salamanca J.I., Fernández A., Rodríguez-Mañas L., Angulo J. (2022). STIM/Orai Inhibition as a Strategy for Alleviating Diabetic Erectile Dysfunction Through Modulation of Rat and Human Penile Tissue Contractility and in vivo Potentiation of Erectile Responses. J. Sex. Med..

[17] Avila-Medina J., Mayoral-González I., Galeano-Otero I., Redondo P.C., Rosado J.A., Smani T. (2020). Pathophysiological Significance of Store-Operated Calcium Entry in Cardiovascular and Skeletal Muscle Disorders and Angiogenesis. Adv. Exp. Med. Biol..

[18] Castillo-Galán S., Arenas G.A., Reyes R.V., Krause B.J., Iturriaga R. (2020). Stim-activated TRPC-ORAI channels in pulmonary hypertension induced by chronic intermittent hypoxia. Pulm. Circ..

[19] Liao Y., Plummer N.W., George M.D., Abramowitz J., Zhu M.X., Birnbaumer L. (2009). A role for Orai in TRPC-mediated Ca^2+^ entry suggests that a TRPC:Orai complex may mediate store and receptor operated Ca^2+^ entry. Proc. Natl. Acad. Sci. USA.

[20] Kochukov M.Y., Balasubramanian A., Noel R.C., Marrelli S.P. (2013). Role of TRPC1 and TRPC3 channels in contraction and relaxation of mouse thoracic aorta. J. Vasc. Res..

[21] Martín-Bórnez M., Galeano-Otero I., Del Toro R., Smani T. (2020). TRPC and TRPV Channels’ Role in Vascular Remodeling and Disease. Int. J. Mol. Sci..

[22] Sung H.H., Choo S.H., Ko M., Kang S.J., Chae M.R., Kam S.C., Han D.H., So I., Lee S.W. (2014). Increased expression of TRPC4 channels associated with erectile dysfunction in diabetes. Andrology.

[23] Ou Z.F., Zhu L.K., Liu Q.W., Jiang J., Jiang R. (2022). Effect of low androgen levels on transient receptor potential channels expression in rat penile corpus cavernosum tissue and its relationship with erectile function. Andrologia.

[24] Jung J.H., Kim B.J., Chae M.R., Kam S.C., Jeon J.H., So I., Chung K.H., Lee S.W. (2010). Gene transfer of TRPC6 (dominant negative) restores erectile function in diabetic rats. J. Sex. Med..

[25] So I., Chae M.R., Kim S.J., Lee S.W. (2005). Lysophosphatidylcholine, a component of atherogenic lipoproteins, induces the change of calcium mobilization via TRPC ion channels in cultured human corporal smooth muscle cells. Int. J. Impot. Res..

[26] Hu D., Ge Y., Cui Y., Li K., Chen J., Zhang C., Liu Q., He L., Chen W., Chen J. (2022). Upregulated IGFBP3 with Aging Is Involved in Modulating Apoptosis, Oxidative Stress, and Fibrosis: A Target of Age-Related Erectile Dysfunction. Oxid. Med. Cell. Longev..

[27] El Assar M., Fernández A., Sánchez-Ferrer A., Angulo J., Rodríguez-Mañas L. (2018). Multivessel analysis of progressive vascular aging in the rat: Asynchronous vulnerability among vascular territories. Mech. Ageing Dev..

[28] Chaudhari S., Mallet R.T., Shotorbani P.Y., Tao Y., Ma R. (2021). Store-operated calcium entry: Pivotal roles in renal physiology and pathophysiology. Exp. Biol. Med..

[29] He L.P., Hewavitharana T., Soboloff J., Spassova M.A., Gill D.L. (2005). A functional link between store-operated and TRPC channels revealed by the 3,5-bis(trifluoromethyl)pyrazole derivative, BTP2. J. Biol. Chem..

[30] Kiyonaka S., Kato K., Nishida M., Mio K., Numaga T., Sawaguchi Y., Yoshida T., Wakamori M., Mori E., Numata T. (2009). Selective and direct inhibition of TRPC3 channels underlies biological activities of a pyrazole compound. Proc. Natl. Acad. Sci. USA.

[31] Miller M., Shi J., Zhu Y., Kustov M., Tian J.B., Stevens A., Wu M., Xu J., Long S., Yang P. (2011). Identification of ML204, a novel potent antagonist that selectively modulates native TRPC4/C5 ion channels. J. Biol. Chem..

[32] Sharma S.H., Pablo J.L., Montesinos M.S., Greka A., Hopkins C.R. (2019). Design, synthesis and characterization of novel N-heterocyclic-1-benzyl-1H-benzo[d]imidazole-2-amines as selective TRPC5 inhibitors leading to the identification of the selective compound, AC1903. Bioorg. Med. Chem. Lett..

[33] Gao Y.Y., Tian W., Zhang H.N., Sun Y., Meng J.R., Cao W., Li X.Q. (2021). Canonical transient receptor potential channels and their modulators: Biology, pharmacology and therapeutic potentials. Arch. Pharm. Res..

[34] Comerma-Steffensen S., Prat-Duran J., Mogensen S., Fais R., Pinilla E., Simonsen U. (2022). Erectile Dysfunction and Altered Contribution of KCa1.1 and KCa2.3 Channels in the Penile Tissue of Type-2 Diabetic db/db Mice. J. Sex. Med..

[35] Yilmaz-Oral D., Sezen S.F., Turkcan D., Asker H., Kaya-Sezginer E., Kirlangic O.F., Kopru C.Z., Elci M.P., Ozen F.Z., Korkusuz P. (2024). Dual Strategy with Adipose-Derived Stem Cells and l-arginine Recovered Cavernosal Functions in a Rat Model of Radical Prostatectomy. Stem Cells Dev..

[36] Ihrig C.M., Montgomery M.M., Nomura Y., Nakano M., Pandey D., La Favor J.D. (2025). Histone deacetylase 6 inhibition prevents hypercholesterolemia-induced erectile dysfunction independent of changes in markers of autophagy. Sex. Med..

[37] Beckmann H., Richter J., Hill K., Urban N., Lemoine H., Schaefer M. (2017). A benzothiadiazine derivative and methylprednisolone are novel and selective activators of transient receptor potential canonical 5 (TRPC5) channels. Cell Calcium.

[38] El Assar M., Angulo J., García-Rojo E., Sevilleja-Ortiz A., García-Gómez B., Fernández A., Sánchez-Ferrer A., La Fuente J.M., Romero-Otero J., Rodríguez-Mañas L. (2022). Early manifestation of aging-related vascular dysfunction in human penile vasculature-A potential explanation for the role of erectile dysfunction as a harbinger of systemic vascular disease. Geroscience.

[39] Hatzimouratidis K., Salonia A., Adaikan G., Buvat J., Carrier S., El-Meliegy A., McCullough A., Torres L.O., Khera M. (2016). Pharmacotherapy for Erectile Dysfunction: Recommendations from the Fourth International Consultation for Sexual Medicine (ICSM 2015). J. Sex. Med..

[40] Rubaiy H.N. (2019). Treasure troves of pharmacological tools to study transient receptor potential canonical 1/4/5 channels. Br. J. Pharmacol..

[41] Zhou Y., Castonguay P., Sidhom E.H., Clark A.R., Dvela-Levitt M., Kim S., Sieber J., Wieder N., Jung J.Y., Andreeva S. (2017). A small-molecule inhibitor of TRPC5 ion channels suppresses progressive kidney disease in animal models. Science.

[42] Xu Y., Ren Y., Zhang J., Niu B., Liu M., Xu T., Zhang X., Shen J., Wang K., Cao Z. (2024). Discovery of pyridazinone derivatives bearing tetrahydroimidazo[1,2-a]pyrazine scaffold as potent inhibitors of transient receptor potential canonical 5 to ameliorate hypertension-induced renal injury in rats. Eur. J. Med. Chem..

[43] Walsh L., Reilly J.F., Cornwall C., Gaich G.A., Gipson D.S., Heerspink H.J.L., Johnson L., Trachtman H., Tuttle K.R., Farag Y.M.K. (2021). Safety and Efficacy of GFB-887, a TRPC5 Channel Inhibitor, in Patients with Focal Segmental Glomerulosclerosis, Treatment-Resistant Minimal Change Disease, or Diabetic Nephropathy: TRACTION-2 Trial Design. Kidney Int. Rep..

[44] Baradaran-Heravi A., Bauer C.C., Pickles I.B., Hosseini-Farahabadi S., Balgi A.D., Choi K., Linley D.M., Beech D.J., Roberge M., Bon R.S. (2022). Nonselective TRPC channel inhibition and suppression of aminoglycoside-induced premature termination codon readthrough by the small molecule AC1903. J. Biol. Chem..

