# Supplementary material for: Targeting TRPC-5 Channel Inhibition to Improve Penile Vascular Function in Erectile Dysfunction
Source: Int J Mol Sci. 2025 Feb 8;26(4):1431. doi: 10.3390/ijms26041431 (PMC11855833; doi:10.3390/ijms26041431)
Supplement: Supplementary file 1 [file ijms-26-01431-s001.zip › Suppl Fig S1 241128.pptx]

## Slide 1
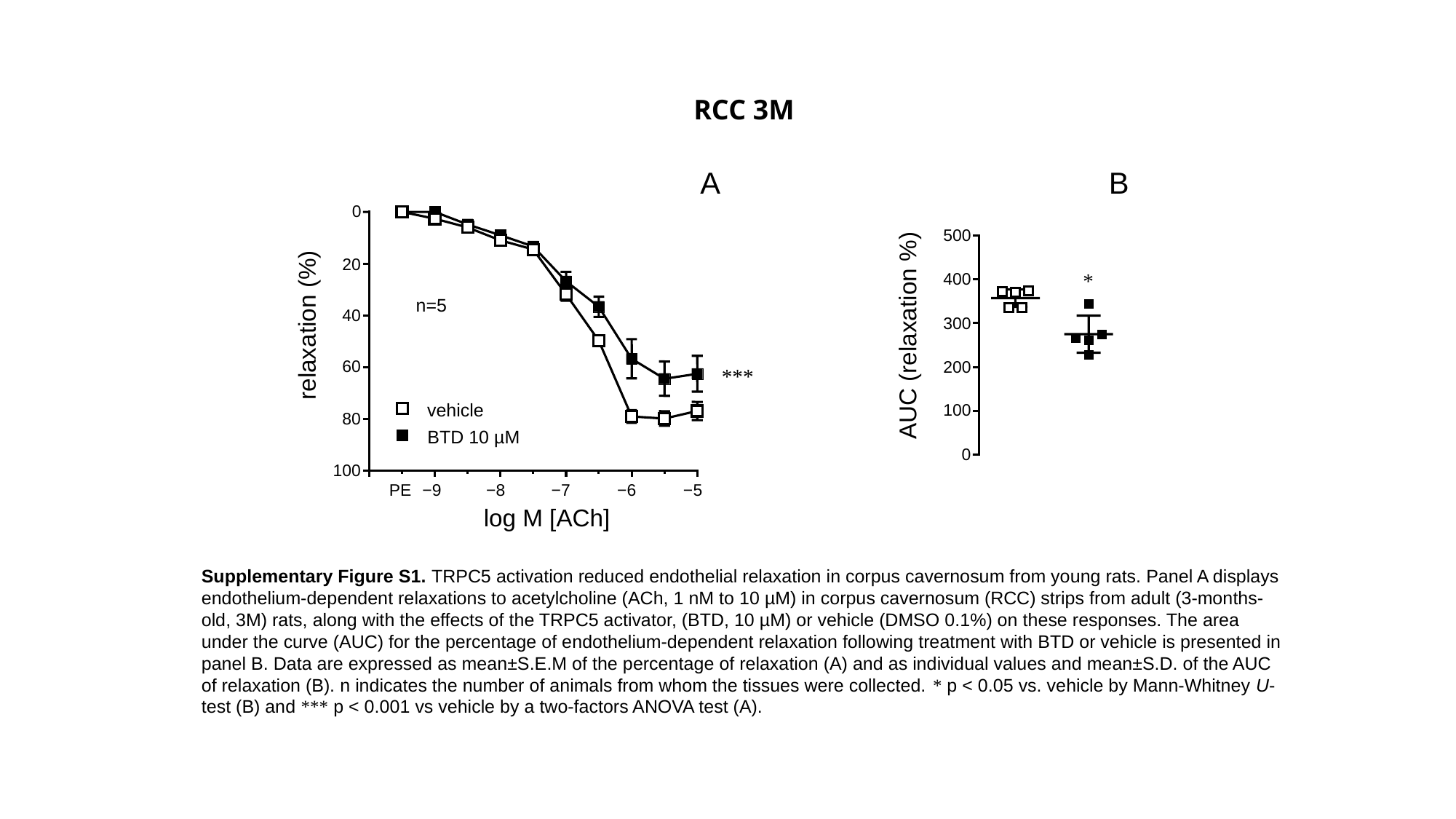

RCC 3M
A
B
0
500
20
*
400
n=5
40
relaxation (%)
300
AUC (relaxation %)
60
200
***
vehicle
100
80
BTD 10 µM
0
100
PE
−9
−8
−7
−6
−5
log M [ACh]
Supplementary Figure S1. TRPC5 activation reduced endothelial relaxation in corpus cavernosum from young rats. Panel A displays endothelium-dependent relaxations to acetylcholine (ACh, 1 nM to 10 µM) in corpus cavernosum (RCC) strips from adult (3-months-old, 3M) rats, along with the effects of the TRPC5 activator, (BTD, 10 µM) or vehicle (DMSO 0.1%) on these responses. The area under the curve (AUC) for the percentage of endothelium-dependent relaxation following treatment with BTD or vehicle is presented in panel B. Data are expressed as mean±S.E.M of the percentage of relaxation (A) and as individual values and mean±S.D. of the AUC of relaxation (B). n indicates the number of animals from whom the tissues were collected. * p < 0.05 vs. vehicle by Mann-Whitney U-test (B) and *** p < 0.001 vs vehicle by a two-factors ANOVA test (A).
