# Supplementary material for: Targeting TRPC-5 Channel Inhibition to Improve Penile Vascular Function in Erectile Dysfunction
Source: Int J Mol Sci. 2025 Feb 8;26(4):1431. doi: 10.3390/ijms26041431 (PMC11855833; doi:10.3390/ijms26041431)
Supplement: Supplementary file 1 [file ijms-26-01431-s001.zip › Suppl Figure S2 250131.pptx]

## Slide 1
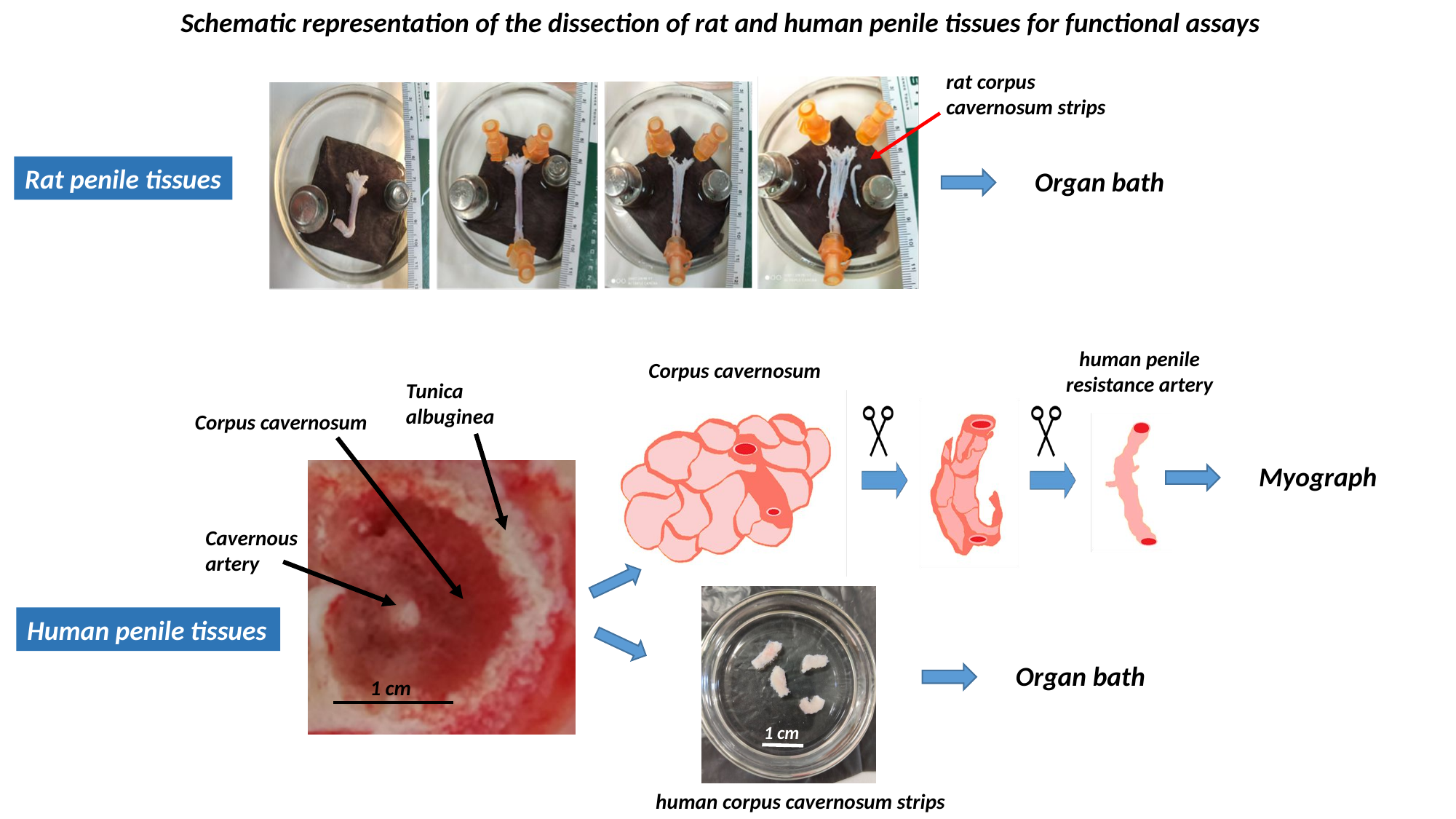

Schematic representation of the dissection of rat and human penile tissues for functional assays
rat corpus cavernosum strips
Rat penile tissues
Organ bath
human penile resistance artery
Corpus cavernosum
Tunica albuginea
Corpus cavernosum
Myograph
Cavernous artery
Human penile tissues
Organ bath
1 cm
1 cm
human corpus cavernosum strips
